# Exploring China stepping into the dawn of chemical pesticide-free agriculture in 2050

**DOI:** 10.3389/fpls.2022.942117

**Published:** 2022-09-09

**Authors:** Xuejiang Wang, Yan Chi, Feng Li

**Affiliations:** Wuzhoufeng Agricultural Science & Technology Co., Ltd., Yantai, China

**Keywords:** autoregressive integrated moving average model, biopesticide, chemical pesticide, compound annual growth rate (CAGR), pesticide-free agriculture

## Abstract

China has implemented a series of policies to reduce the usage of chemical pesticides to maintain food production safety and to reduce water and soil pollution. However, there is still a huge gap in developing biological pesticides to replace chemical agents or managing pests to prevent crop production loss. It is necessary to predict the future use of chemical pesticides and to exploit the potential ways to control pests and crop diseases. Pesticide usage is affected by seasonal changes and analyzed by using a seasonal autoregressive integrated moving average (ARIMA) model (a statistical model that predicts future trends using time-series data). The future development of biopesticides in China was predicted using the compound annual growth rate (CAGR), which is calculated *via* the equation [(Final value/Starting value)^1/years^ – 1] according to the annual growth rate of target products over time. According to the reducing trend of pesticide and biological pesticide usage annually, China is predicted possibly step into the era of pesticide-free agriculture in 2050 based on the analysis of the ARIMA model. With CAGR calculation, China will produce from 500 thousand to one million tons of biopesticides in 2050, which can meet the need to replace chemical pesticides in agriculture to prevent the present crop production loss. To achieve the goal, China still has the greatest challenges to develop biopesticides and use various strategies to control pest and crop diseases. China may step into the dawn of chemical pesticide-free agriculture in 2050 if biopesticides can be developed smoothly and pests can be controlled well using various strategies.

## Introduction

As an important means of production, pesticides play an important role in controlling pest damage and increasing crop yield. However, the long-term application of a large number of chemical pesticides not only brings serious environmental pollution problems but also affects the quality and safety of agricultural products and biodiversity and affects the sustainable development of human society. Cyfluthrin is in a group of man-made insecticides and is widely used in homes, outdoors, and in agriculture to effectively control phytophagous insect pests (Rolim et al., 2021). β-Cyfluthrin induces acute arrhythmic cardiotoxicity through interaction with NaV1.5, and ranolazine reverses the phenotype (da Silva et al., 2022). Fomesafen is an organic compound used as a herbicide (Meng et al., 2019). Water-solubility of fomesafen leads to a potential risk to groundwater, and it has been reported that the death of fish is related to fomesafen (Dong et al., 2019). Pyraclostrobin is an agricultural pesticide product used to kill most fungi, including blights, mildews, molds, and rusts (Yu et al., 2022), and has characterized lethal toxicity (Huang et al., 2021). Pesticide consumption primarily threats biodiversity and causes its significant decline (Parween and Jan, 2019).

By linking pesticide usage reports from Chinese statistical yearbooks, this study shows that pesticides adversely affect health outcomes *via* drinking water exposure. A difference-in-difference-in-differences framework to compare health outcomes between people who drink surface water and groundwater in regions with different intensities of rice pesticide use before and after 1 year indicated that a 10% increase in rice pesticide use unfavorably altered a key medical disability index (activities of daily living) by 1% for rural residents (Lai, 2017). It has been reported that more than 150 million miles of China’s farmland are contaminated (Mishra et al., 2016). Since the discovery of dichloro-diphenyl-trichloroethane (DDT) and hexachlorocyclohexane (BHC), their excessive and persistent application has led to severe environmental pollution and human health risks (Huang and Lu, 2021). Even so, the application of chemical pesticides is still the major method to protect crops from yield loss (Sharma et al., 2019). In contrast, biopesticide sources are easily obtained in nature, are naturally biodegradable, act in different modes, and possess less toxicity to live organisms (Thakur et al., 2020). It is imperative to reduce the amount of chemical pesticides and replace them with biological pesticides (Ruiu, 2018).

To feed 20% of the world’s population with only 7% of the world’s arable land, China has a heavy reliance on chemical pesticides to maintain high-level crop yields and long-term capital gains (Li et al., 2020). China has become the world’s largest consumer of chemical pesticides. Unfortunately, in the past few years, pesticide pollution in the air, water, and soil and deaths caused by pesticides have been serious in China (Zhang et al., 2019; Karunarathne et al., 2020). Pesticide poisoning often happens when chemical pesticides are used to control a pest and affects humans (Mostafalou and Abdollahi, 2013), wildlife (Köhler and Triebskorn, 2013), plant (Parween et al., 2016), or beneficial insects (Siviter and Muth, 2020). Although the effects of pesticide poisoning have been remarkably controlled, the number of fatal cases is still high due to the largest population in China. It is necessary to explore the future change under the new policies. Chemical pesticide consumption is affected by seasonal varieties (Gao et al., 2019). According to the reducing trend of pesticide usage annually, a seasonal autoregressive integrated moving average (ARIMA) model (a statistical model that predicts future trends by using time-series data) will be suitable to predict chemical pesticide consumption in the future. Reducing chemical pesticide consumption has become a solid goal shared by many countries and a major issue in public policies due to its high risk to human life and negative impacts on the environment. Pesticide-free agriculture has become a new paradigm for research (Jacquet et al., 2022).

As early as 2015, China launched the “Action Plan to Realize Zero Growth in Pesticide Use by 2020” and completed this goal ahead of schedule in 2017 (Shuqin and Fang, 2018). With the support and encouragement of national policies, a large number of high-efficiency, low-toxicity, low-residue, and low-risk pesticides have also been quickly created, applied, and promoted, and biological pesticides have gradually risen and received more and more attention. There were 115 biological pesticide active ingredients, and more than 3,800 registered formulated products in China before 2018, including five different types of biopesticides, including microbial pesticides, botanical pesticides, biochemical pesticides, natural enemies, and agricultural antibiotics (Lihong, 2018). The top five microbial pesticides in China include *Bacillus thuringiensis* (Ahmed et al., 2019), *Bacillus subtilis* (Guo et al., 2019), *Helicoverpa armigera nuclear polyhedrosis virus* (Lu et al., 2022), *Metarhizium anisopliae* (Peng et al., 2021), and *Paenibacillus polymyxa* (Cheng et al., 2022) according to the production output.^1^ To protect human health and achieve high-level crop yields, biopesticides should be further developed in the future. Its changes were also calculated in this study to explore its possible products meeting sustainable agriculture development.

## Methods

### Data collection and analysis

The data for pesticide consumption in China were obtained from the National Bureau of Statistics,^2^ the previous publication (Zhang et al., 2011; Sharma et al., 2019; Li et al., 2021), and Internet data^3^ (Supplementary Table 1).

### A seasonal autoregressive integrated moving average model for forecasting chemical pesticide consumption in China in 2050

**FIGURE 1:**
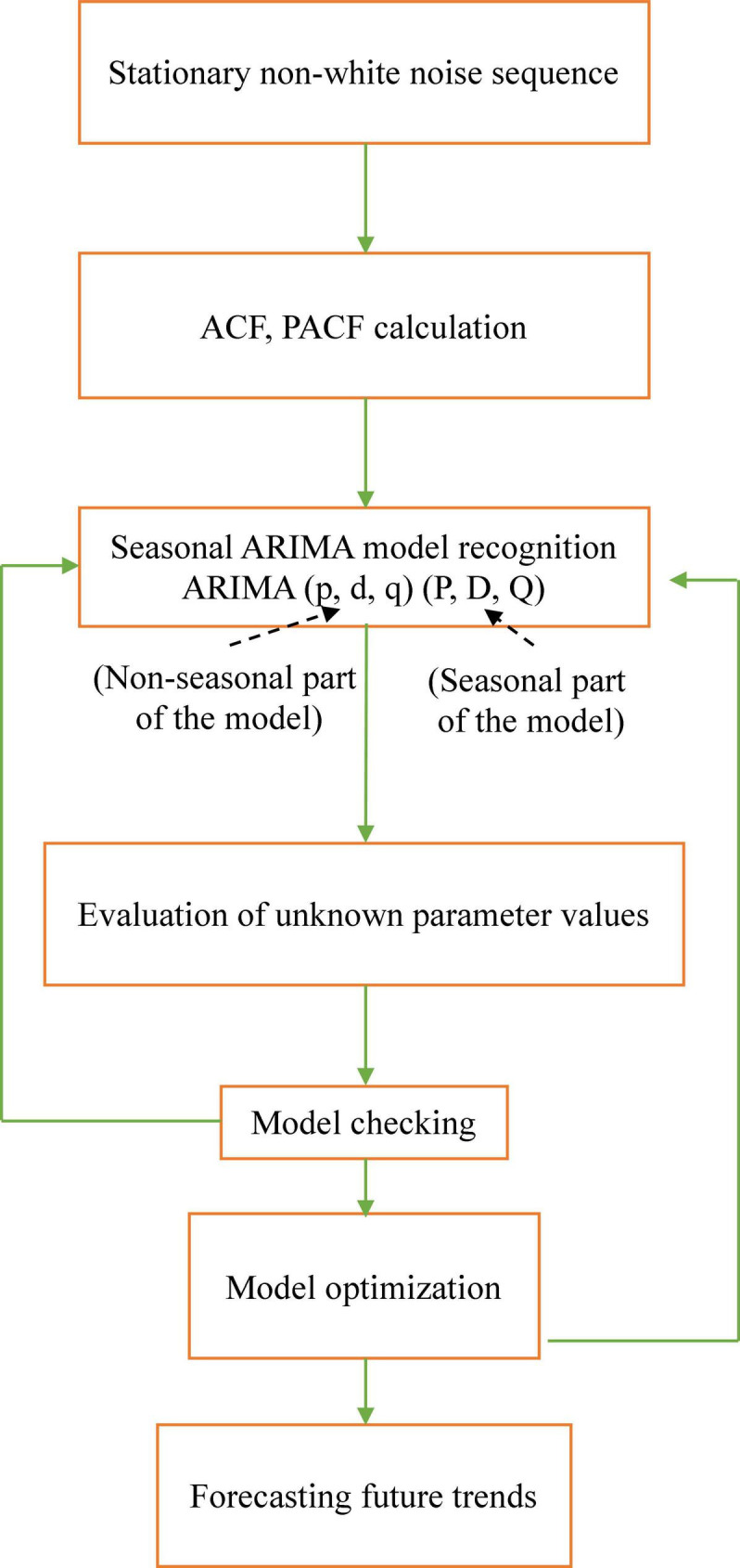
Flow chart of seasonal ARIMA model steps. Agricultural chemical pesticides forecasting in China was performed using seasonal ARIMA (p,d,q) × (P,D,Q)S model with p = nonseasonal autoregressive (AR) order, d = nonseasonal differencing, q = nonseasonal moving average (MA) order, P = seasonal AR order, D = seasonal differencing, Q = seasonal MA order, and S = time span of repeating seasonal pattern (Abdul-Aziz et al., 2013). ARIMA, autoregressive integrated moving average; ACF, autocorrelation function; PACF, partial autocorrelation.

Time series analysis is a vital branch of statistics, has the basic principles of mathematical statistics, and can support a series of scientifically dynamic data processing approaches (Xu and Chen, 2021). It is a scientific method with strong applicability that has developed rapidly based on probability theory and mathematical statistics and is supported by computer applications. A time series is a sequence of random variables formed by variables in the order of time intervals. A large number of statistical indicators in the fields of nature, social economy, etc., are counted by year, quarter, month, or day. Pesticide use has striking seasonal variability (Larsen et al., 2019) and will be fitted to be analyzed using a seasonal model. Time series analysis of variables is often carried out using the seasonal ARIMA approach (Dimri et al., 2020). ARIMA model is a statistical model that is autoregressive if it forecasts future values according to previous values. The seasonal ARIMA model extends an ARIMA model by taking seasonality into account (Supplementary material). Agricultural chemical pesticide forecasting in China was performed using an approach to the seasonal ARIMA (p,d,q) × (P,D,Q)S model according to a previous report with slight modification (Figure 1; Ahamed et al., 2020).

### The compound annual growth rate for forecasting future growth rates of biopesticide consumption in China until 2050

Besides the above harmful effects on human health and threats to human life, the costs to develop a synthetic agricultural chemical has been increased more than 200-fold from 1956 to 2016 (McDougall, 2016). The high levels of chemical pesticide production further aggregately pollute soil, drinking water, and environment and cause more serious life-threatening to human beings. Therefore, it is critical to look for more efficient ways to minimize the harmful environmental impact. Biopesticides come into play and provide an eco-friendly alternative to chemical pesticides. To better understand the development of biopesticides, it will be helpful to predict the biopesticide market in the future. Compound annual growth rate (CAGR) is often used way to calculate the annual growth rate of target products over time according to Equation 1. The future development of biopesticides in China is calculated according to the presently reported data (Supplementary Table 2). Based on the previous reports, the amounts of biopesticides occupy 10–20% of that of chemical pesticides (Zhang et al., 2011). Mycopesticides accounted for 10% of the global biopesticide market in 2016 (Zaki et al., 2020). The market value of biological additives has been US$ 3 billion every year in recent years with an increase at an average annual growth rate of 20%. By 2015, China had registered 4,293 biological pesticides with 112 active ingredients (Wang et al., 2018). The Chinese biopesticide market is expected to grow at a CAGR of 4.6% during the period 2020–2025.^4^ Thus, the increasing rate will be arranged between 4.6 and 20%.


(1) $$\text{CAGR}={\left(\mathrm{Final}\,\mathrm{value}/\mathrm{Starting}\,\mathrm{value}\right)}^{1/\text{years}}-1$$

## Results and discussion

### China achieved zero growth in chemical pesticide consumption since 2014

**FIGURE 2:**
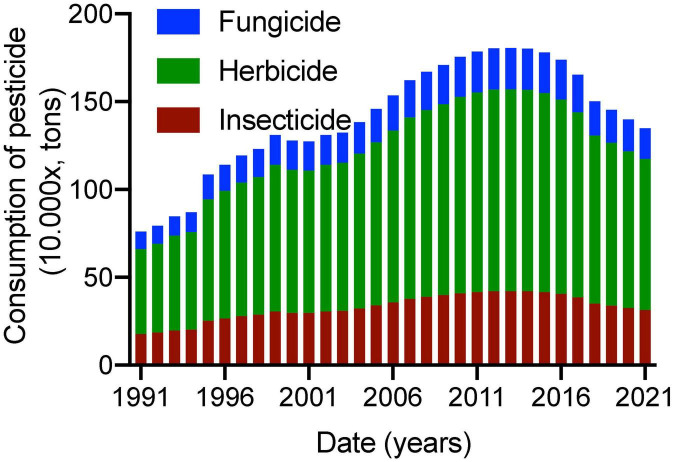
Chemical pesticide consumption in China from 1991 to 2021. Pesticides include fungicide, herbicide, and insecticide.

Chemical pesticide consumption in China from 1991 to 2021 mainly includes fungicides, herbicides, and insecticides, and among them, herbicides occupy more than 60% of all pesticides (Figure 2). Chemical pesticide consumption in China has increased from 76 million tons in 1991 to 146 million tons in 2006 (Sharma et al., 2019). China had negative growth in chemical pesticide consumption in 2015 and zero growth in chemical pesticide consumption since 2014 (Figure 2), which is earlier than the previously reported schedule in 2017 and the policy of 2020 (Shuqin and Fang, 2018). In 2018, chemical pesticide consumption in China reached approximately 1.5 million tons. As of 2019, chemical pesticide consumption in China reduced to 1.39 million tons (Li et al., 2021) and still is the major contributing country to pesticide usage in the world (Sharma et al., 2019).

### China may step into the era of chemical pesticide-free agriculture in 2050

**FIGURE 3:**
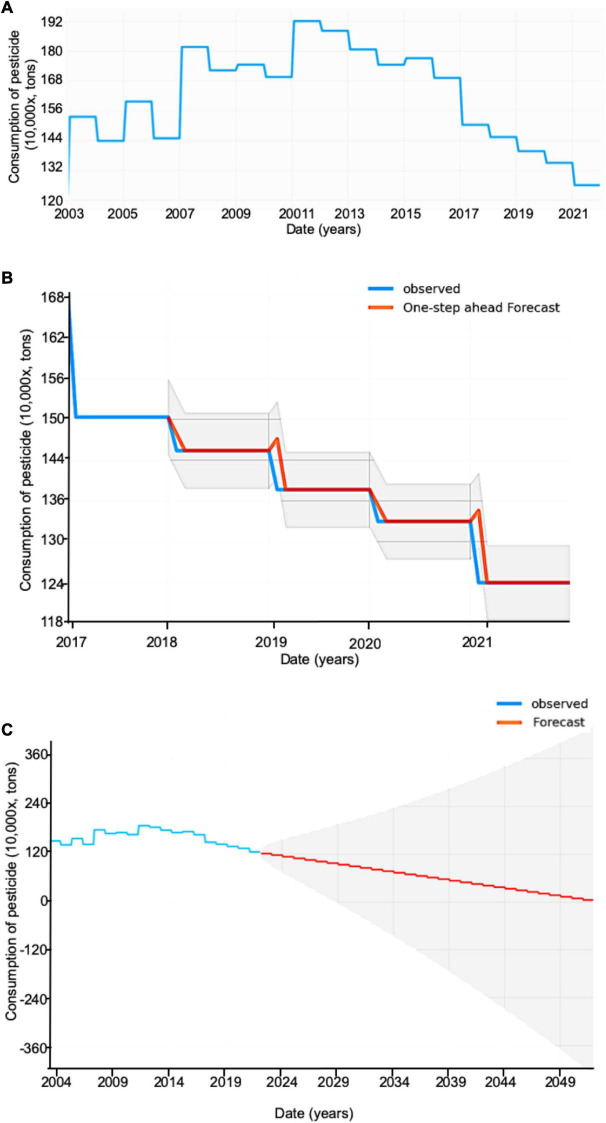
A seasonal ARIMA model for time series forecasting in the chemical pesticide consumption in China in 2050. **(A)** The chemical pesticide consumption in China from 2003 to 2021. **(B)** Forecasting data are approved using the observed data. **(C)** The chemical pesticide consumption will reach zero by 2050 based on the seasonal ARIMA model. Gray areas indicate the observed maximum positive and negative relationship between chemical pesticide consumption and date (years). ARIMA, autoregressive integrated moving average.

Time series analysis and diagnostics show that the chemical pesticide consumption has significant seasonal parameters in the trend for the ARIMA model (Figure 3A). A seasonal model will be helpful to predict future changes in pesticide consumption. From 2003 to 2021, the consumption of pesticides was lowest in 2003, increased in 2004, and reached the highest level in 2012. The consumption of pesticides showed a declined trend until 2021 since 2012, and the reduction in pesticide consumption was reduced by more than 30% (Figure 3A). Pesticide use has striking seasonal variability (Larsen et al., 2019), and thus a seasonal model may be fitted to analyze the changing pattern. Forecasting data of chemical pesticide consumption from 2018 to 2021 are well tested with perfect fit using the observed data (Figure 3B), suggesting that the model will be suitable for forecasting the chemical pesticide consumption level in China. The seasonal ARIMA model predicts that chemical pesticide consumption will reach zero levels in China in 2050 based on the previous data (Figure 3C). The results suggest that China may step into the era of chemical pesticide-free agriculture in 2050.

### The development of biopesticide will meet the demand for sustainable agricultural development in China

**FIGURE 4:**
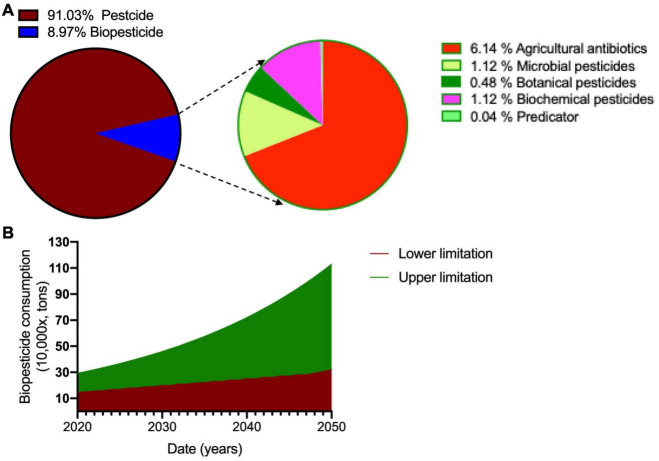
The development of biopesticide in China. **(A)** The proportion of biopesticides to the proportion of chemical pesticides in China in 2020. **(B)** The development of biopesticide in China until 2050 based on CAGR calculation. CAGR, compound annual growth rate.

In 2006, the total biopesticide consumption reached 145 thousand tons, with nearly 10% of total pesticides (Figure 4A; Zhang et al., 2011). Among all biopesticides, agricultural antibiotics occupy the major proportion and are followed by microbial pesticides, biochemical pesticides, and botanical pesticides. Predicator occupies the smallest proportion among all biopesticides (Figure 4A). China will produce from 300 thousand to one million tons of biopesticides in 2050 (Figure 4B), which will meet the needs of chemical pesticide-free agriculture to maintain high yield and quality crop production.

China has achieved zero growth in chemical pesticide consumption since 2014, which meets China’s policy basis (Shuqin and Fang, 2018). There is a significant decrease in chemical pesticide consumption from 2013 to 2022 (Figure 2). The remarkable reduction in chemical pesticide consumption is closely associated with agricultural safety policy improvement in China. Agricultural policy of the XXI century in product quality and safety will be founded to make sure obligatory traceability for potentially unsafe products in China (Yu, 2021).

Chemical pesticide consumption will reach zero level in China in 2050 (Figure 3C), which is accordant with the hope of chemical pesticide-free agriculture in Europe in 2050 (Mora et al., 2021). Diversification will be a potential approach to reinforce biological regulations to control pests and diseases *via* biodiversity. By 2050, a sustainable and virtuous circle of agricultural ecosystems will be achieved with 30% of multifunctional, safe, and efficient fertilizers, 30% of biopesticides, 2% of the light energy utilization rate of C3 crops such as rice and wheat, and 30% of the comprehensive utilization rate of the soil, fertilizer, and water.

Pesticide consumption is subject to changing climatic scenarios and thus has seasonal changing characteristics. Therefore, a seasonal ARIMA model was used to predict the chemical pesticide consumption in China until 2050 (Figure 3). We predict that China will possibly step into the era of pesticide-free agriculture in 2050. However, to achieve high yield and quality crop production, China still has the greatest challenges to develop biopesticides with the largest population in the world. It is very vital to perfect the following jobs for biopesticide production.

## Conclusion and future directions

Many plants contain a large amount of brassinosteroids. As an environmentally friendly substance, the use of brassinosteroids not only reduces the negative impact of a large number of chemical pesticides on the ecological environment but also plays a role in killing pests and reducing the impact of diseases and insect pests on the crop (Liu et al., 2021; Bakshi et al., 2022). When preventing and controlling crop diseases and insect pests, staff should use botanical pesticides reasonably to achieve the purpose of effectively controlling diseases and insect pests. Since this type of pesticide is mainly based on the extracted plant physical components, the use of this pesticide to carry out the control of crop diseases and insect pests is relatively less polluting to the environment than traditional chemical pesticides (Bringel and Couée, 2018; Serra et al., 2020). In addition, the price of this pesticide is cheap, which reduces farmers’ capital investment in agricultural production, reduces pesticide residues in crops, improves the control effect, and lays a solid foundation for the healthy growth of crops.

The biological metabolism method is one of the most effective pest control methods in the current crop pest control work, as the microbial control method has the characteristics of low side effects, is harmless to the human body, and causes no environmental pollution (Dara et al., 2019; Mascarin et al., 2019). The use of microbial control methods to carry out pest control is mainly based on inhibiting pests from eating bacteria and fungi and using the microbial preparations formed by them to carry out pest control. Some important plant pests are attacked by virus diseases. For instance, the baculoviruses can attack most leaf-feeding caterpillars and sawflies, which damage most land plants. The appropriate use of Nosema locustae in plant protection is as a grasshopper management tool, for long-term suppression (Cranshaw and Klein, 2020). Therefore, more efforts are needed to improve the application and research of microbial control methods in the prevention and control of crop diseases and insect pests.

To ensure the quality and safety of agricultural products and reduce the residues of traditional chemical pesticides, the technology and products of new crop biological disaster prevention and control will inevitably become the focus of innovation in the future. China will greatly rely on scientific and technological progress, including biological control, plant immunity, and pheromone prevention. Cytokinins are plant hormones, which affect not only plant growth, development, and physiology but also interact with plant pathogens by improving the defense and susceptibility of plants against bacterial and fungal pathogens and pest insects via the improvement of plant immunity (Akhtar et al., 2020). Mating disruption with sex pheromones is an effective and safe approach for controlling insect pests. However, the high cost of chemically synthesized pheromones limited their usage. The model yeasts can be considered for producing pest pheromones and pheromone precursors (Holkenbrink et al., 2020). In terms of biological pesticides, we will create high-titer engineered strains to optimize microbial fermentation and stabilize expression technology. China still needs to develop new products, such as RNA interference agents, pheromone inducers, and control agents, and establish a biocontrol microbial resource library.

New light sources and semichemical application technologies for insect trapping and killing will be developed (Abbas et al., 2019; Holkenbrink et al., 2020). Pest communication regulation technology and sterile irradiation technology will be developed to reduce environmental risks, improve prevention and control effects, and guarantees the green management of crop biological disasters.

Integrated Pest Management (IPM) is an environmentally friendly, common sense approach to control pests and focuses on ecological aspects of pest management (Bell and Bennett, 2022; Jowett et al., 2022). IPM is the potential way to address pest management, including the development of management strategies, the management of information and making timely decisions, and the dissemination or sharing of information (Dara, 2019). Climate change, environmental pollution, and exhaustion of natural resources are the noticeable challenges for sustainable crop development and environmental management in modern agriculture. Bio-based IPM is one of the effective ways to control pests with environmentally benign, effective, and economically viable (Fahad et al., 2021). IPM is the most innovative treatment, used fewer pesticides, and may be an economic way (Larsen et al., 2019). Prioritizing components of the package of IPM and better economic benefits can be obtained (Yadav et al., 2019). The analysis with an IPM model with Leslie–Gower type and ratio-dependent functional response show that a cost minimization model can be created to determine the optimal control level *via* the order-1 periodic solution (Xu et al., 2018).

Replacing synthetic insecticides with transgenic crops for pest control is a also potential way of pest management (Taylor et al., 2021). Plant-derived protease inhibitors (PIs) are a promising defensin for increasing crop production and improving insect pest management. These vital genes of insects can be used *via* the gene-editing technique, such as CRISPR/Cas9. This research is developing differently in silico and modern molecular biology techniques to apply PIs to control insect pests (Singh et al., 2020). Biological control agents and pheromones have limited utilization in pest management. Biotechnology has provided effective new approaches to control insect pests, such as synthetic biology (Mateos Fernández et al., 2022).

Unfortunately, there are still some potential conflicts of interest involved in the original reporting of pesticide use data in China. Biopesticide development is a potential way to replace the use of chemical pesticides but the high-cost production of biopesticides still limits its usage (Damalas and Koutroubas, 2018). Furthermore, long-term usage of chemical pesticides will induce the resistance of pests to the agents, which reduced the effectiveness of biopesticides (Delnat et al., 2019). Therefore, there are still some challenges to the development of high-effective biopesticides.

China will possibly step into the era of pesticide-free agriculture in 2050 according to the analysis based on a seasonal ARIMA model. However, to prevent crop production loss, China still has the greatest challenges to develop biopesticides and biological techniques and maintain biodiversity.

## Data availability statement

The raw data supporting the conclusions of this article will be made available by the authors, without undue reservation.

## Author contributions

XW and YC were involved in the initial conceptualization and design of this manuscript. All authors performed the experiment and analyzed the data and wrote and provided revisions to the manuscript, contributed to this study and approved the submitted version.

## Footnotes

1
https://www.reach24h.com/en/news/industry-news/agrochemical/compliance-suggestions-for-china-biological-pesticides.html

2
http://www.stats.gov.cn/

3
https://www.statista.com/statistics/863620/pesticide-consumption-volume-in-china/

4
https://www.mordorintelligence.com/industry-reports/china-biopesticides-market-industry

## Supplementary material

Supplementary Material The calculation of the seasonal autoregressive integrated moving average (ARIMA) model.
