# Supplementary material for: Exploring China stepping into the dawn of chemical pesticide-free agriculture in 2050
Source: Front Plant Sci. 2022 Sep 9;13:942117. doi: 10.3389/fpls.2022.942117 (PMC9504061; doi:10.3389/fpls.2022.942117)
Supplement: Supplementary Material — The calculation of the seasonal autoregressive integrated moving average (ARIMA) model. [file Data_Sheet_1.docx]

Table S1: Chemical pesticide consumption in China from 1991 to 2021. Pesticides include fungicide, herbicide and insecticide (10,000 tons).

| Date (years) | Insecticide | Herbicide | Fungicide | Total |
| --- | --- | --- | --- | --- |
| 1991 | 17.7313 | 48.41482 | 9.893 | 76.03912 |
| 1992 | 18.5235 | 50.5779 | 10.335 | 79.4364 |
| 1993 | 19.7817 | 54.01338 | 11.037 | 84.83208 |
| 1994 | 20.2943 | 55.41302 | 11.323 | 87.03032 |
| 1995 | 25.3271 | 69.15494 | 14.131 | 108.61304 |
| 1996 | 26.5853 | 72.59042 | 14.833 | 114.00872 |
| 1997 | 27.8435 | 76.0259 | 15.535 | 119.4044 |
| 1998 | 28.7056 | 78.37984 | 16.016 | 123.10144 |
| 1999 | 30.5696 | 83.46944 | 17.056 | 131.09504 |
| 2000 | 29.824 | 81.4336 | 16.64 | 127.8976 |
| 2001 | 29.7075 | 81.1155 | 16.575 | 127.398 |
| 2002 | 30.5696 | 83.46944 | 17.056 | 131.09504 |
| 2003 | 30.87716 | 84.309224 | 17.2276 | 132.413984 |
| 2004 | 32.2938 | 88.17732 | 18.018 | 138.48912 |
| 2005 | 34.01567 | 92.878838 | 18.9787 | 145.873208 |
| 2006 | 35.8121 | 97.78394 | 19.981 | 153.57704 |
| 2007 | 37.8159 | 103.25526 | 21.099 | 162.17016 |
| 2008 | 38.9576 | 106.37264 | 21.736 | 167.06624 |
| 2009 | 39.843 | 108.7902 | 22.23 | 170.8632 |
| 2010 | 40.9614 | 111.84396 | 22.854 | 175.65936 |
| 2011 | 41.6371 | 113.68894 | 23.231 | 178.55704 |
| 2012 | 42.0798 | 114.89772 | 23.478 | 180.45552 |
| 2013 | 42.1264 | 115.02496 | 23.504 | 180.65536 |
| 2014 | 42.01689 | 114.725946 | 23.4429 | 180.185736 |
| 2015 | 41.5439 | 113.43446 | 23.179 | 178.15736 |
| 2016 | 40.55365 | 110.73061 | 22.6265 | 173.91076 |
| 2017 | 38.5615 | 105.2911 | 21.515 | 165.3676 |
| 2018 | 35.0432 | 95.68448 | 19.552 | 150.27968 |
| 2019 | 33.9248 | 92.63072 | 18.928 | 145.48352 |
| 2020 | 32.62 | 89.068 | 18.2 | 139.888 |
| 2021 | 31.455 | 85.887 | 17.55 | 134.892 |

Table S2: Biopesticide production in China from 2010 to 2025 (10,000 tons).

| Date (years) | Biopesticide production | Increasing rate |
| --- | --- | --- |
| 2010 | 10.13 |  |
| 2011 | 12.91 | 27.44% |
| 2012 | 13.62 | 5.5% |
| 2013 | 14.86 | 9.1% |
| 2014 | 18.35 | 23.49% |
| 2015 | 21.76 | 18.58% |
| 2016 | 25.03 | 15.03% |
| 2017 | 27.53 | 9.99% |
| 2018 | 30.14 | 9.48% |
| 2019 | 33.61 | 11.51% |
| 2020 | 37.07 | 10.29% |
| 2021 | 40.94 | 10.44% |
| 2022 | 45.35 | 10.77% |
| 2023 | 50.11 | 10.5% |
| 2024 | 55.41 | 10.58% |
| 2025 | 61.28 | 10.59% |

Note: China Economic Industry Information Research Network (http://www.cniir.com). Zhang Xiling, & Si Xuezian. (2011). Opportunities and challenges for the development of biological pesticides. New Countryside (CN23-1552/S), (12), 24-24.
